# Green Synthesis and Characterization of *Rosa roxburghii* Tratt.-Mediated Gold Nanoparticles for Visual Colorimetric Assay of Tiopronin

**DOI:** 10.3390/nano15191513

**Published:** 2025-10-03

**Authors:** Dan Liu, Shilan Feng

**Affiliations:** 1 School of Medical Health and Wellness, Shaanxi Energy Institute, Xianyang 712000, China; liudan_cpu@163.com; 2 School of Pharmacy, Lanzhou University, Lanzhou 730000, China

**Keywords:** green synthesis, gold nanoparticles, colorimetric sensor, tiopronin

## Abstract

This study used *Rosa roxburghii* Tratt. crude extract (RR) as a reducing, stabilizing, and modifying agent for the green synthesis of gold nanoparticles (RR-AuNPs) via the one-pot method for the first time and established a novel colorimetric sensor for detecting tiopronin. Initially, RR-AuNPs with a uniform particle size and stable dispersion were prepared using the reducing property of RR. Upon the introduction of tiopronin, the drug binds to the surface of RR-AuNPs through Au-S bonds and hydrogen bonds, inducing a significant aggregation of RR-AuNPs. The absorbance of the RR-AuNP solution exhibited a linear relationship with the tiopronin concentration in the range of 0.17 μM to 16.67 μM (y = 1.9157 − 0.0972x), with a detection limit of 0.19 μM. The colorimetric sensor was successfully applied to detect tiopronin in urine samples. Compared with other detection methods, this approach is simple to operate and has a high sensitivity, a wide linear range, and a low detection limit.

## 1. Introduction

Tiopronin, N-(2-mercaptopropionyl) glycine, is a sulfhydryl-containing drug similar in properties to penicillamine. It is widely used in the treatment of cystinuria and rheumatoid arthritis and also has the effects of liver protection and detoxification [1]. However, the use of tiopronin can cause skin allergies or adverse reactions in the digestive system, such as allergic reactions, urticaria, allergic purpura, anaphylactic shock, abdominal pain, elevated liver enzymes, submandibular gland enlargement, parotid gland enlargement, etc. [2].

At present, many methods for the detection of tiopronin have been reported, such as high-performance liquid chromatography, ultraviolet–visible (UV-vis) spectrophotometry, liquid chromatography–ultraviolet (LC-UV) detection, etc. [3,4,5,6,7]. Although these instrumental methods have the advantages of a high sensitivity and accuracy, due to the particularity of large instruments, they have the disadvantages of high costs and a complex pretreatment process. There is an urgent need to develop a rapid, sensitive, and portable method for the detection of tiopronin.

Gold nanoparticles (AuNPs) have been widely applied in fields such as optics, chemistry, electricity, and medicine due to their unique physical, chemical, and optical properties [8]. AuNPs exhibit distinct localized surface plasmon resonance (LSPR) characteristics, which manifest as a strong characteristic absorption peak in their ultraviolet–visible (UV-vis) absorption spectra. The position of this LSPR absorption peak depends on factors such as the size, shape, composition, and interparticle distance of AuNPs, collectively determining their solution color. AuNPs with sizes ranging from 10 to 20 nm typically show a characteristic absorption peak at 520 nm, presenting a unique red color. As the diameter of AuNPs increases, the SPR peak gradually shifts to longer wavelengths. When targets are introduced into AuNPs, the position of the characteristic absorption peak, absorbance value, and solution color of AuNPs change with the concentration of the targets, enabling their application in colorimetric sensing [9]. Colorimetric sensors based on AuNPs can detect various substances, including metal ions, drugs, peptides, proteins, etc. [10,11]. Compared with traditional detection methods, LSPR-based gold nanoparticle sensors offer advantages such as convenience, rapidity, accuracy, and high sensitivity, playing a crucial role in analytical detection.

By controlling the dosage and type of reducing agents, the type of ligands, and synthesis conditions, researchers have prepared AuNPs with different particle sizes modified with different ligands. At present, the methods for preparing AuNPs are mainly divided into physical synthesis methods and chemical synthesis methods: physical synthesis methods refer to converting bulk gold into nanoscale particles by means of high energy consumption. Common methods include electrical dispersion, vacuum evaporation, vapor-phase synthesis, soft landing, etc. [12,13]. Physical synthesis methods are complex to carry out and have a low yield, and it is difficult to control particle formation [14]. Classical chemical synthesis methods use chloroauric acid as raw material, use reducing agents (such as borohydride, citric acid, etc.) to reduce gold nanoparticles, and use stabilizers (such as trisodium citrate dihydrate, sulfur ligands, etc.) to stabilize the nanoparticles, so as to control the growth rate, size, or shape of the nanoparticles [15,16]. The toxic reagents used in traditional chemical synthesis methods not only limit their applications in the biomedical field, but also may cause harm to human health and the environment. Green synthesis methods, which are characterized by their low costs, simplicity, and low toxicity, have been widely studied in recent years [17,18,19].

Green-synthesized AuNPs exhibit good biocompatibility, high stability, and excellent catalytic, antibacterial, and antioxidant properties, further enhancing their application value in various fields [20,21,22]. Bacteria, proteins, plant extracts, fruits, and agricultural waste have been widely employed as reducing agents for the green synthesis of AuNPs. Among them, plant extracts, due to their bioactive components such as polyphenols, flavonoids, amino acids, and proteins, can rapidly synthesize size-controllable and monodisperse AuNPs, making them the optimal choice for the large-scale green synthesis of AuNPs. During the synthesis of gold nanoparticles, they can serve not only as reducing and stabilizing agents but also as modifiers on the surface of gold nanoparticles [23]. *Rosa roxburghii* Tratt., the fresh or dried fruit of *Rosa roxburghii* in the Rosaceae family, is known as the “king of vitamin C”. The crude extract of *Rosa roxburghii* Tratt. (RR) is rich in active components such as polyphenols, organic acids, and polysaccharides, and it has superior antioxidant activity [24]. In this paper, RR was successfully used for the first time for the green synthesis of RR-functionalized gold nanoparticles (RR-AuNPs), in which RR played multiple roles as a reducing agent, modifier, and stabilizer.

Based on the green-synthesized RR-AuNPs, this study designed a convenient, sensitive, and highly selective colorimetric sensor for a visual colorimetric assay of tiopronin and applied it to the detection of tiopronin in urine samples. The green-synthesized RR-AuNPs have a uniform size and good dispersibility, with a characteristic absorption peak at 530 nm and a particle size of approximately 24.30 nm. Tiopronin binds to the surface of RR-AuNPs through Au-S bonds and hydrogen bonds, causing a significant aggregation of RR-AuNPs. A simple, sensitive, and visual colorimetric method for the detection of tiopronin was established, with a linear range of 0.17 μM to 16.67 μM and a detection limit of 0.19 μM.

## 2. Experimental Sections

### 2.1. Instruments and Materials

The instruments used included a scanning electron microscope (SEM) (Sigma 360, ZEISS, Jena, Germany); zeta potential analyzer (Malvern Zetasizer Nano ZS90, Malvern Panalytical, Malvern, UK); Fourier transform infrared spectrometer (FT-IR) (Thermo Fisher Scientific Nicolet iS20, Waltham, MA, USA); transmission electron microscope (TEM) (JEOL JEM-F200, JEOL, Tokyo, Japan); UV-vis spectrophotometer (UV-1800PC-DS2, Shanghai Mepuda Instruments Co., Ltd., Shanghai, China); freeze dryer (LGJ-12, Sihuan Furui Science and Technology Co., Ltd., Beijing, China); and nanoparticle size analyzer (Winner 802, Jinan Weina Particle Instruments Co., Ltd., Jinan, China).

The materials included chloroauric acid trihydrate (HAuCl_4_·3H_2_O) (Sigma-Aldrich, St. Louis, MO, USA); *Rosa roxburghii* Tratt. crude extract (Xi’an Shengqing Biotechnology Co., Ltd., Xi’an, China); and tiopronin (Aladdin Biochemical Technology Co., Ltd., Shanghai, China). Urine samples were collected from healthy volunteers. Experimental water was Watsons distilled water.

### 2.2. Green Synthesis of RR-AuNPs

A volume of 60 μL of 10% HAuCl_4_·3H_2_O solution was dissolved in 60 mL of ultrapure water and stirred at room temperature. A total of 0.04 g of RR was dissolved in 10 mL of ultrapure water. We quickly added the prepared RR solution to the HAuCl_4_·3H_2_O solution. We stirred the entire reaction for 10 min. During the reaction, the color of the solution gradually changed from light yellow to wine red, and RR-AuNPs were finally synthesized. The synthesized RR-AuNPs had a particle size of approximately 24.30 nm and a characteristic absorption wavelength of 530 nm. Their extinction coefficient is 7.66 × 10^9^ L/mol/cm [25]. According to the Lambert–Beer law, the molar concentration of RR-AuNPs was calculated to be approximately 0.23 nmol/L (A = Kbc and c = A/Kb, where A is the absorbance value, K is the extinction coefficient, and b is the thickness of the absorption cell).

### 2.3. Optimization of Synthesis Conditions

To optimize the synthesis conditions, the effects of varying reducing agent concentrations (1 mg/mL, 2 mg/mL, 4 mg/mL, 8 mg/mL, and 10 mg/mL), reaction temperatures (25 °C, 40 °C, 60 °C, 80 °C, and 100 °C), and reaction times (1 min, 10 min, 20 min, 40 min, and 60 min) on the green synthesis of gold nanoparticles were investigated. The optimal synthesis conditions were determined by visually observing the color changes of the RR-AuNP solution and comparing the characteristic absorption peaks in UV-vis absorption spectra.

### 2.4. Characterization of RR-AuNPs

The particle size of the solution was recorded using a nanoparticle size analyzer. The zeta potential of the solution was measured with a zeta potential analyzer. The morphology of particles was observed via SEM. The UV-vis absorption spectra of the solution were recorded using a UV-vis spectrophotometer. The structure of particles and energy dispersive spectroscopy (EDS) were characterized by TEM. The surface chemical functional groups and molecular structure of particles were analyzed by FT-IR.

### 2.5. Investigation of RR-AuNPs’ Stability

The effect of pH (1 to 14) on the stability of RR-AuNPs was studied by adjusting the solution pH with 0.5 M NaOH and 0.5 M HCl. The effect of salt concentration (0 nM to 200 nM) on the stability of RR-AuNPs was investigated by adjusting the solution salt concentration with 0.1 M NaCl. The stability of RR-AuNPs was studied at temperatures ranging from 25 °C to 100 °C and at different incubation times (0 min, 10 min, 20 min, 40 min, and 60 min). The color changes of RR-AuNP solution were monitored. The UV-vis absorption spectra and the absorbance at 530 nm of RR-AuNPs were recorded.

### 2.6. Visual Colorimetric Detection of Tiopronin

Based on previous studies, the experiments were conducted at a constant temperature of 25 °C, with the reaction system maintained at pH = 5, and a fixed incubation time of 10 min (no additional salt was added to the system). A series of tiopronin solutions with gradient concentrations were added to freshly prepared RR-AuNP solution (3000 μL, 0.23 nmol/L) and mixed. The final concentrations of tiopronin were 0.17 μmol/L, 0.50 μmol/L, 1.67 μmol/L, 3.33 μmol/L, 5.00 μmol/L, and 16.67 μmol/L, respectively. The color changes of the solution were visually observed, and the UV-vis absorption spectra were recorded. A linear curve was established with the absorbance at 530 nm (A) as the ordinate and the tiopronin concentration as the abscissa.

### 2.7. Selectivity Investigation

To ensure its practical application value, the selectivity of the colorimetric sensor was investigated. Different interfering substances were added to 3000 μL of 0.23 nmol/L RR-AuNPs, with the final concentration of each interfering substance set to 6.67 μM. The detection procedure was the same as that in Section 2.6.

### 2.8. Real Sample Detection

Urine samples from human subjects were subjected to simple pretreatment: the urine samples were filtered through a 0.45 μm membrane and diluted 10-fold with ultrapure water for standby. A certain concentration of tiopronin was added to each pretreated urine sample. The entire pretreatment process was simple and took no more than 10 min. The detection procedure was the same as that in Section 2.6.

## 3. Results and Discussion

### 3.1. Green Synthesis of RR-AuNPs

**Scheme 1:**
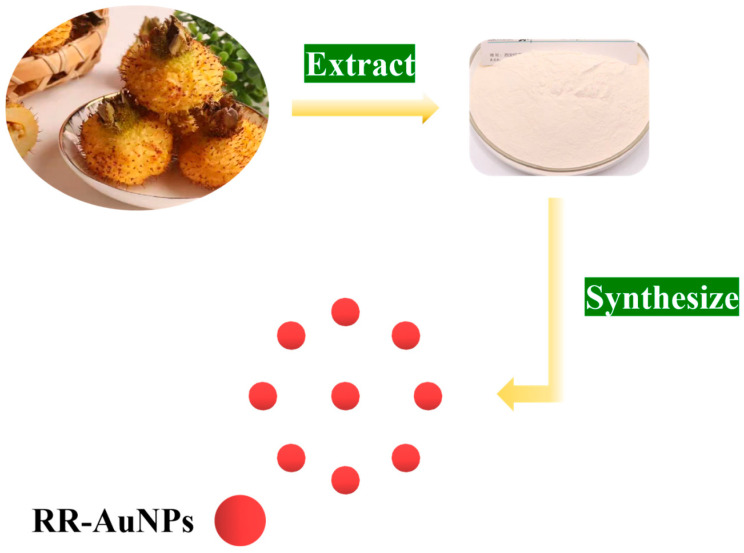
Green-synthesized RR-AuNPs.

Scheme 1 illustrates the green synthesis process of RR-AuNPs. The crude extract of *Rosa roxburghii* Tratt. is a complex mixture rich in vitamin C, polyphenols, superoxide dismutase, and other components, with a total content exceeding 10%, exhibiting strong reducibility. Therefore, in the presence of the crude *Rosa roxburghii* Tratt. extract, free AuCl_4_^−^ ions in the solution could be reduced to Au(0), which aggregated into AuNPs.

**Figure 1:**
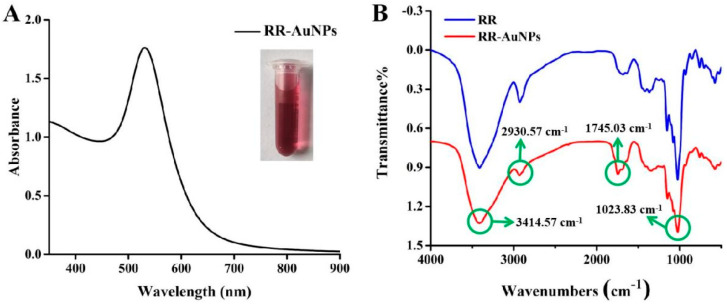
(**A**) The UV-vis absorption spectra and a photograph of RR-AuNPs. (**B**) The FT-IR spectra of RR and RR-AuNPs.

As shown in Figure 1A, the synthesized RR-AuNP solution presented a wine-red color. When detected by a UV-vis spectrophotometer, an asymmetric and narrow characteristic absorption peak was observed at 530 nm. These results preliminarily indicated that the formed RR-AuNPs had good dispersibility. According to the FT-IR spectrum in Figure 1B, the absorption peaks of -OH, -C-H, -C=O, and -C-O appeared at 3414.57 cm^−1^, 2930.57 cm^−1^, 1745.03 cm^−1^, and 1023.83 cm^−1^, respectively. This confirmed that the crude extract of *Rosa roxburghii* Tratt. was successfully functionalized on the surface of RR-AuNPs. In summary, the crude extract of *Rosa roxburghii* Tratt. played an important role as a reducing agent, stabilizer, and modifier in the green synthesis of RR-AuNPs. Subsequently, multiple characterization methods were used to detect RR-AuNPs and study their stability under external environments, confirming that the green-synthesized RR-AuNPs had good dispersibility, a uniform particle size, and excellent stability.

**Figure 2:**
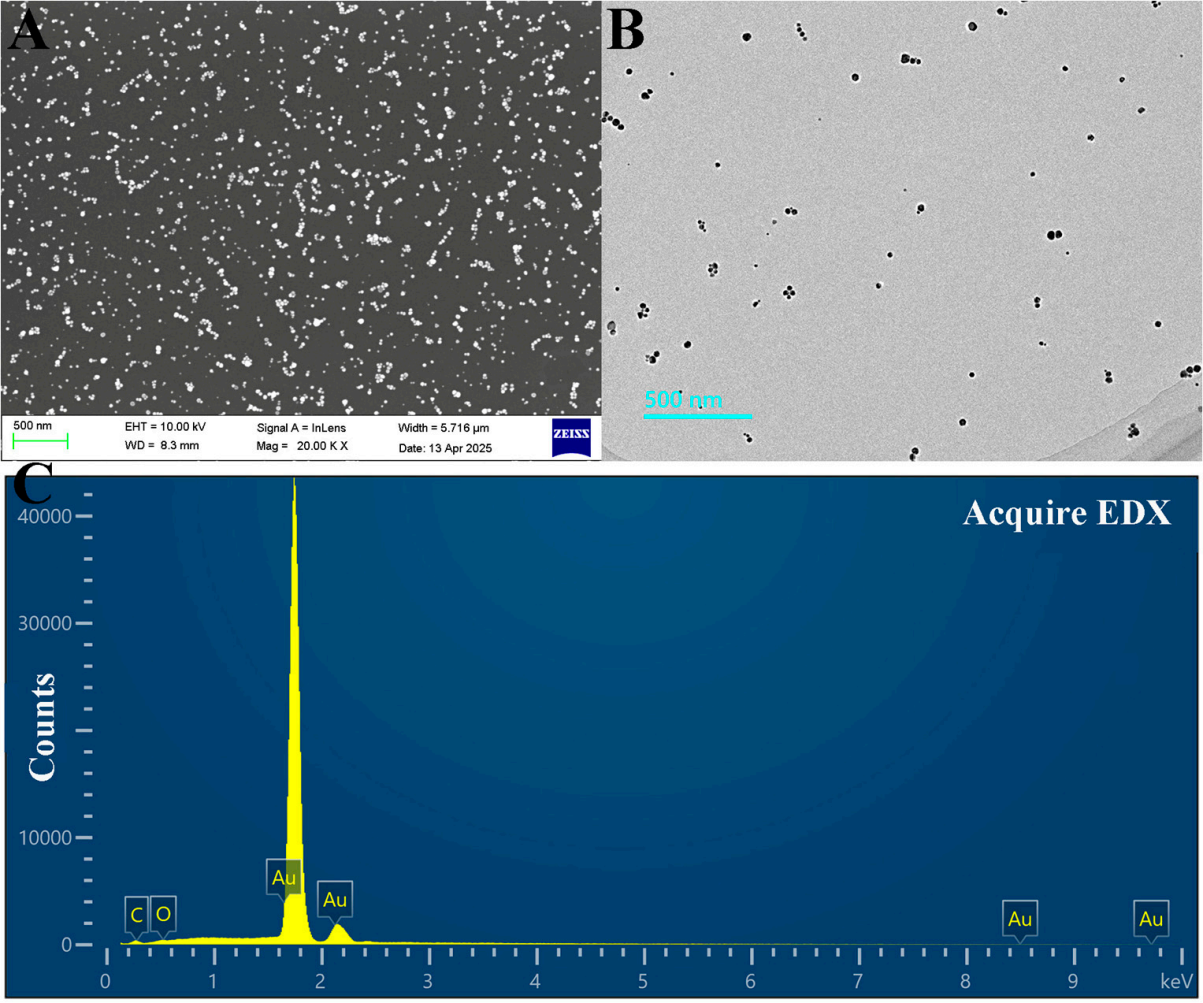
(**A**) The SEM image of RR-AuNPs. (**B**) The TEM image of RR-AuNPs. (**C**) The EDS image of RR-AuNPs.

Figure 2A,B show that most of the RR-AuNPs exhibited a spherical or near-spherical morphology, with good dispersion in the solution. As shown in Figure S1, the average particle size of RR-AuNPs was 24.30 nm with a standard deviation of 7.61 nm, which was calculated from a histogram of the TEM image. The energy-dispersive X-ray spectroscopy (EDS) in Figure 2C further confirmed the presence of carbon, oxygen, and gold atoms, which were primarily derived from the crude *Rosa roxburghii* Tratt. extract. Combined with the results from Figure 1, these characterizations collectively validated the successful synthesis of RR-AuNPs.

**Figure 3:**
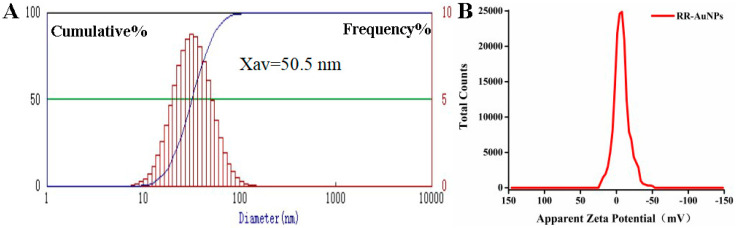
(**A**) The particle size distribution of RR-AuNPs. (**B**) The zeta potential of RR-AuNPs.

As shown in Figure 3, the dynamic light scattering (DLS) results showed that the hydrodynamic diameter of the RR-AuNPs was approximately 50.5 nm, and the zeta potential was about −7.12 mV.

### 3.2. Synthesis Conditions of RR-AuNPs

The particle size, morphology, and aggregation state of gold nanoparticles determine their properties and applications. Therefore, the key issue in their preparation is how to obtain monodisperse, uniformly sized, and stable gold nanoparticles. To determine the synthesis conditions, the color changes of the RR-AuNP solutions synthesized with different reductant concentrations, reaction temperatures, and reaction times were observed, and their UV-vis spectra were measured. As shown in Figure S2, when the concentration of *Rosa roxburghii* Tratt. crude extract ranged from 1 mg/mL to 10 mg/mL, the color of the RR-AuNP solution deepened with increasing concentration, and the absorbance at 530 nm first increased and then decreased. At an extract concentration of 4 mg/mL, the solution displayed a wine-red color and the most intense UV absorption peak, suggesting that this concentration was optimal for the synthesis of RR-AuNPs. Figure S3 showed that varying the reaction time from 1 min to 60 min caused no significant changes in the solution color or UV absorption peak, confirming that reaction time has no obvious effect on the RR-AuNPs’ synthesis. As can be seen from Figure S4, when the reaction temperature increased from 25 °C to 100 °C, the solution color deepened continuously while the UV absorption peak weakened. As shown in Figure S5, the SEM images of RR-AuNPs synthesized at 25 °C, 40 °C, 60 °C, 80 °C, and 100 °C were compared. The results clearly demonstrated that no anisotropic structures (such as triangular or hexagonal nanosheets) formed as the reaction temperature increased, and RR-AuNPs exhibit varying degrees of aggregation with rising temperature. This confirmed that the changes in the color and UV characteristic absorption peaks of the RR-AuNP solution were attributable to varying degrees of aggregation. Based on the above results and considering cost-effectiveness, the optimal synthesis conditions were determined as follows: a *Rosa roxburghii* Tratt. crude extract concentration of 4 mg/mL, reaction time of 10 min, and reaction temperature of 25 °C.

### 3.3. Stability of RR-AuNPs

According to previous studies, RR-AuNPs exhibit a uniform particle size, good dispersibility, and a characteristic absorption peak at 530 nm at room temperature. Given the significant influence of external conditions on the properties of RR-AuNPs, their stability under different conditions was further investigated by adjusting the pH, standing time, temperature, and salt concentration of the solution. As shown in Figures S6 and S7, when the pH value varied from 1 to 14, the color and characteristic absorption peak of the RR-AuNP solution showed almost no change, illustrating that the RR-AuNPs could remain stable under both acidic and alkaline conditions. The stability of the RR-AuNP solution over a certain period (0 min to 60 min) was studied. As shown in Figure S8, RR-AuNPs could maintain a stable dispersion state within this period. The interference of temperature on the stability of RR-AuNPs was further explored. Figure S9 showed the good stability of RR-AuNPs in the temperature range of 25 °C to 80 °C. When the temperature rose to 100 °C, the absorbance value of RR-AuNPs at 530 nm decreased slightly, but the solution color showed no obvious change. The effect of different salt concentrations on the stability of RR-AuNPs was investigated. The results presented in Figure S10 indicated that when the salt concentration increased from 0 nM to 200 nM, the color and characteristic absorption peak of the RR-AuNP solution remained almost unchanged, proving that RR-AuNPs could maintain stable dispersion within a certain salt concentration range. It can be seen from the above that RR-AuNPs can maintain a stable dispersion state within a wide range of conditions.

### 3.4. Visual Colorimetric Detection of Tiopronin

**Figure 4:**
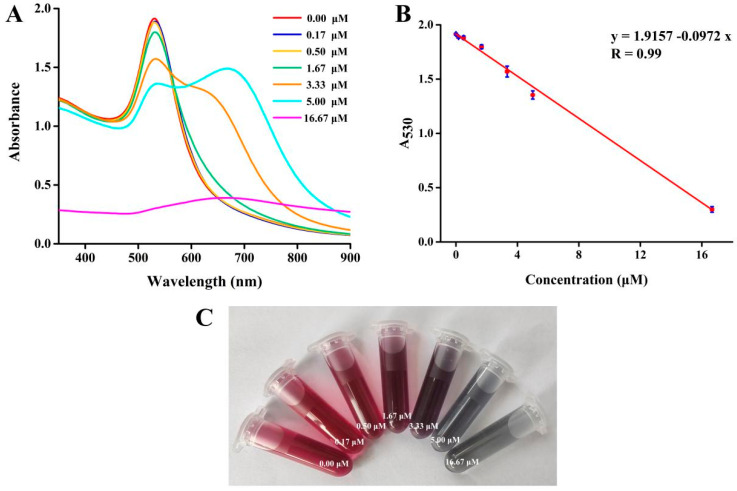
(**A**) The UV-vis absorption spectra of RR-AuNPs in the presence of increasing concentrations of tiopronin. (**B**) The linear calibration curve of absorbance values at 530 nm versus the concentration of tiopronin. (**C**) The photograph of RR-AuNPs in the presence of increasing concentrations of tiopronin.

The colorimetric sensing capability of RR-AuNPs for tiopronin was investigated under optimal conditions. Figure 4 shows photographs of solution colors and the UV-vis absorption spectra of RR-AuNP solutions with different tiopronin concentrations. The characteristic UV absorption peak of the blank RR-AuNP solution appeared at 530 nm. As the tiopronin concentration increased from 0.17 μM to 16.67 μM, the absorbance at 530 nm decreased, and the color gradually changed from wine red to gray. A good linear relationship was observed between the absorbance at 530 nm and tiopronin concentration within the range of 0.17~16.67 μM. The calibration curve was established with the absorbance at 530 nm (A_530nm_) as the ordinate and tiopronin concentration as the abscissa: y (A_530nm_) = 1.9157 − 0.0972x, with a correlation coefficient of 0.99 (where A_530nm_ is the absorbance at 530 nm when different tiopronin concentrations are added, and x is the tiopronin concentration). The limit of detection (LOD) was calculated as 0.19 μM using the formula LOD = 3σ/s, where σ represents the blank standard deviation and s represents the slope of the linear regression equation. Table 1 compares the linear ranges and LOD values for tiopronin detection by different methods, demonstrating that the detection system in this study had a broad linear range and a low LOD. Additionally, most reported methods suffer from disadvantages such as high costs and complicated sample preparation. Thus, a sensitive, rapid, and visual colorimetric detection method was developed in this work, holding promise for practical sample detection.

### 3.5. Colorimetric Detection Mechanism of RR-AuNPs

**Figure 5:**
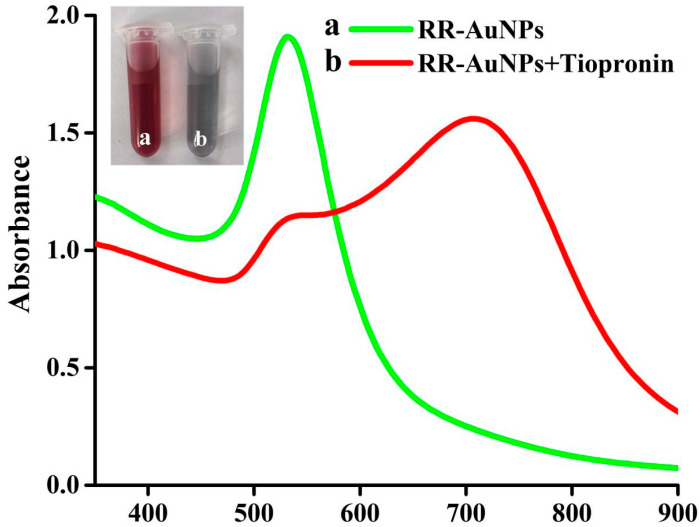
The UV-vis absorption spectra and a photograph of RR-AuNPs in the absence of tiopronin and in the presence of tiopronin.

**Scheme 2:**
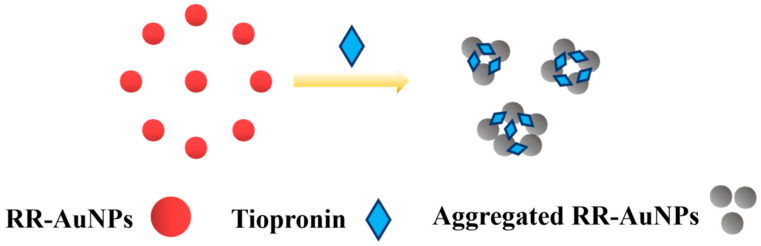
The mechanism of visual colorimetric assay of tiopronin by RR-AuNPs.

The colorimetric detection mechanism of tiopronin by RR-AuNPs was investigated. As shown in Figure 5, the RR-AuNP solution exhibited a wine-red color with a characteristic absorption peak at 530 nm in the absence of tiopronin. However, in the presence of tiopronin, the absorbance at 530 nm decreased accompanied by a red shift, and the color changed from wine red to gray. Because the *Rosa roxburghii* Tratt. crude extract contains biological molecules such as polyphenols, polysaccharides, and proteins, these molecules act as natural ligands adsorbed on the surface of gold nanoparticles during the synthesis of RR-AuNPs, endowing the RR-AuNPs’ surface with polar groups such as phenolic hydroxyl (-OH), carbonyl (C=O), hydroxyl (-OH), amino (-NH_2_), and carboxyl (-COOH). More recently, it has been experimentally confirmed that the binding of organothiols to gold is a result of RS-H or S-S bond cleavage followed by the formation of an Au-S bond. The Au-S bond exhibits dual covalent–ionic properties. The dissociation energy of the Au-S bond is approximately 300 kJ/mol, exhibiting significantly stronger interactions than hydrogen bonds (approximately 10~40 kJ/mol) and van der Waals forces (2~20 kJ/mol). Weak Au(I)-Au(I) interactions contribute negligibly to system stability and can be disregarded in thiol–gold adsorption systems. Experimentally, X-ray photoelectron spectroscopy (XPS), scanning tunneling microscopy (STM), transmission electron microscopy (TEM), and thermogravimetric analysis (TGA) all effectively validate the formation of the Au-S bond [26,27,28,29]. Hence, as illustrated in Scheme 2, the proposed interaction mechanism between RR-AuNPs and tiopronin is as follows: ① Tiopronin contains a thiol group (-SH), which binds to the surface of RR-AuNPs via Au-S bonds. When multiple tiopronin molecules adsorb onto the surface of gold nanoparticles, and the thiol groups of tiopronin simultaneously bind to different gold nanoparticles, they link multiple gold nanoparticles together, inducing the aggregation of the nanoparticles. ② The carboxyl group (-COOH) of tiopronin can form hydrogen bonds with hydroxyl (-OH), carboxyl (-COOH), and carbonyl (-C=O) groups on the RR-AuNP surface. Although weaker than gold–sulfur bonds, these cumulative hydrogen bonds synergize with gold–sulfur interactions to drive extensive RR-AuNP aggregation, which is consistent with our observation that both thiol and carboxyl groups are critical for aggregation.

**Figure 6:**
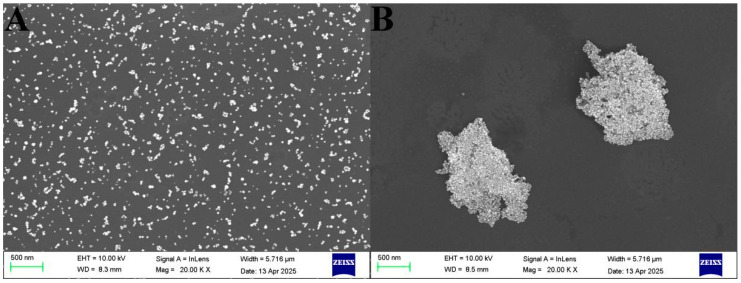
The SEM image of RR-AuNPs in the absence of tiopronin (**A**) and in the presence of tiopronin (**B**).

**Figure 7:**
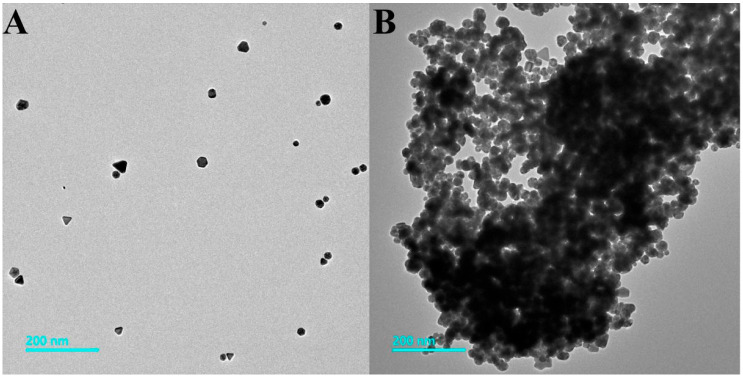
The TEM image of RR-AuNPs in the absence of tiopronin (**A**) and in the presence of tiopronin (**B**).

**Figure 8:**
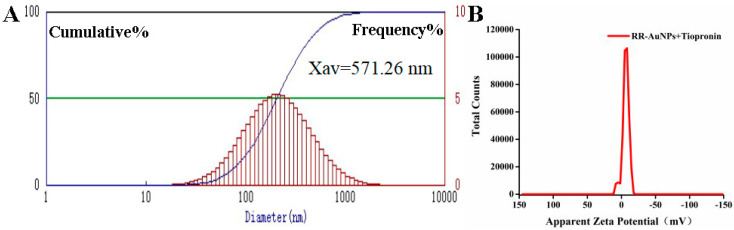
The particle size distribution (**A**) and the zeta potential (**B**) of RR-AuNPs in the presence of tiopronin.

To further explore its mechanism of action, we also compared the SEM images of RR-AuNPs in the absence and presence of tiopronin. The results are shown in Figure 6. When tiopronin was absent, there was no obvious aggregation of RR-AuNPs. In the presence of tiopronin, RR-AuNPs formed large-scale aggregation. As shown in Figure 7, the TEM images of the RR-AuNPs also showed that the RR-AuNPs were in a stable dispersed state in the absence of tiopronin, while the RR-AuNPs aggregated in large numbers in the presence of tiopronin. The zeta potential and hydrodynamic diameters of RR-AuNPs were also compared. As shown in Figure 8A, the particle size of RR-AuNPs significantly increased in the presence of thiopronin, further indicating the formation of large-scale aggregates. Figure 8B shows that the zeta potential of the RR-AuNPs remained unchanged regardless of the presence of thiopronin. Table 2 presents the specific values for the zeta potential and hydrodynamic diameters of the RR-AuNPs before and after the addition of thiopronin, with the results corresponding to those in Figure 8. A comprehensive analysis of the SEM, TEM, and particle size results further confirmed the previous hypothesis on the mechanism of RR-AuNPs for the detection of tiopronin.

### 3.6. Selectivity Investigation

**Figure 9:**
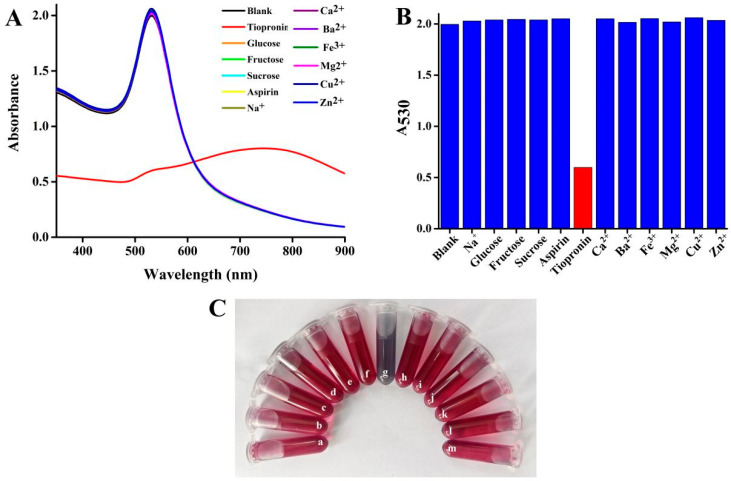
(**A**) The UV-vis absorption spectra of the selectivity of RR-AuNPs (from a to m: blank, Na^+^, glucose, fructose, sucrose, aspirin, tiopronin, Ca^2+^, Ba^2+^, Fe^3+^, Mg^2+^, Cu^2+^, and Zn^2+^, with all of the concentrations being 6.67 μM). (**B**) The histogram corresponding to (**A**). (**C**) The photograph of RR-AuNPs corresponding to (**A**) (from a to m: blank, Na^+^, glucose, fructose, sucrose, aspirin, tiopronin, Ca^2+^, Ba^2+^, Fe^3+^, Mg^2+^, Cu^2+^, and Zn^2+^, with all of the concentrations being 6.67 μM).

To evaluate the selectivity of the colorimetric sensor for tiopronin, several common interfering substances were detected, such as glucose, fructose, sucrose, aspirin, Na^+^, Ca^2+^, Ba^2+^, Fe^3+^, Mg^2+^, Cu^2+^, Zn^2+^, etc. The final concentration of all substances was determined to be 6.67 μM, and the detection procedure was the same as before. As shown in Figure 9, compared with other substances, the absorbance value of RR-AuNPs decreased significantly after adding tiopronin, and the solution color changed from wine red to gray, demonstrating that tiopronin can cause the massive aggregation of RR-AuNPs. However, the absorbance values and solution colors of other interfering substances showed no obvious changes. Therefore, the sensor exhibited good selectivity for tiopronin, furthering its application in actual sample detection.

### 3.7. Real Sample Detection

**Figure 10:**
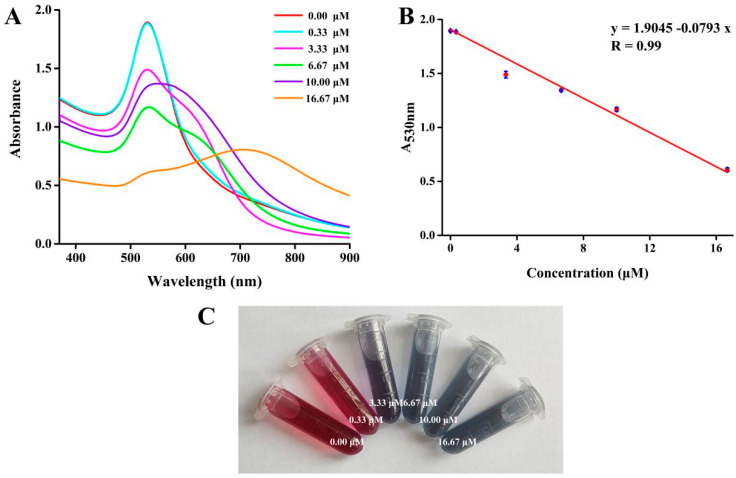
(**A**) The UV-vis absorption spectra of RR-AuNPs in urine samples with increasing tiopronin concentrations. (**B**) The linear calibration curve of absorbance values at 530 nm versus the concentration of tiopronin in urine samples. (**C**) A photograph of RR-AuNPs in urine samples with increasing tiopronin concentrations.

The sensor was further applied to urine samples. Different concentrations of tiopronin were added to the prepared samples. As shown in Figure 10, with the concentration of tiopronin increasing from 0.33 μM to 16.67 μM, the color of the RR-AuNP solution gradually changed from wine red to gray, and the absorbance at 530 nm gradually decreased. A linear curve was established with the absorbance value (A) at 530 nm as the ordinate and the concentration of tiopronin as the abscissa: y (A_530nm_) = 1.9045 − 0.0973x, with a correlation coefficient R of 0.99 (where A_530nm_ is the absorbance of the solution at a wavelength of 530 nm when different concentrations of tiopronin are added, and x is the concentration of tiopronin). The above results confirmed the potential application value of the sensor in real sample detection.

## 4. Conclusions

In this study, a novel colorimetric sensor based on RR-AuNPs was designed. The sensor significantly improved the sensitivity for tiopronin detection, and our findings were as follows: ① For the first time, *Rosa roxburghii* Tratt. crude extract was used for the one-pot green synthesis of RR-AuNPs with a uniform particle size and stable dispersion. ② Tiopronin bound to the surface of RR-AuNPs through Au-S bonds and hydrogen bonds, causing the massive aggregation of RR-AuNPs. ③ A simple, sensitive, and visual colorimetric detection method for tiopronin was established and successfully applied to real sample detection.

## Figures and Tables

**Table 1:** A comparison of different assay methods for tiopronin.

Various Detection Methods	Linear Range	LOD	Ref
Novel nanoprobe	50–400 μM	146 μM	[3]
Chemiluminescence	0.5 μM–3000 μM	0.2 μM	[4]
High-performance liquid chromatography	0.25~24.54 μM	−	[5]
Sulphur- and nitrogen-doped carbon dots	4–18 μM	3.8 μM	[6]
Liquid chromatography with ultraviolet detection	1~200 μM	0.5 μM	[7]
This work	0.17 μM~16.67 μM	0.19 μM	−

**Table 2:** Comparison of zeta potential and hydrodynamic diameters of RR-AuNPs before and after the addition of tiopronin.

	RR-AuNPs	RR-AuNPs + Tiopronin
zeta potential	−7.12 mV	−6.36 mV
hydrodynamic diameters	50.5 nm	571.26 nm

## Data Availability

The original contributions presented in this study are included in the article/Supplementary Materials. Further inquiries can be directed to the corresponding author.

## Supplementary Materials

The following supporting information can be downloaded at: https://www.mdpi.com/article/10.3390/nano15191513/s1, Figure S1. Size distribution histograms of RR-AuNPs from TEM images. (A) Count-based size distribution histogram with Gaussian fitting. (B) Percentage - based size distribution histogram with Gaussian fitting. Figure S2. The UV-Vis absorption spectra and photograph (A) and the absorbance trends at 530 nm (B) for RR-AuNPs with different concentrations of reducing agent. Figure S3. The UV-Vis absorption spectra and photograph (A) and the absorbance trends at 530 nm (B) of RR-AuNPs at different reaction times. Figure S4. The UV-Vis absorption spectra and photograph (A) and the absorbance trends at 530 nm (B) of RR-AuNPs at different reaction temperatures. Figure S5. The SEM image of RR-AuNPs at 25 °C (A), at 40 °C (B), at 60 °C (C), at 80 °C (D) and at 100 °C (E). Figure S6. The UV-Vis absorption spectra (A), the absorbance trends at 530 nm (B) and photograph (C) of RR-AuNPs at pH 1–7. Figure S7. The UV-Vis absorption spectra (A), the absorbance trends at 530 nm (B) and photograph (C) of RR-AuNPs at pH 8–14. Figure S8. The UV-Vis absorption spectra (A), the absorbance trends at 530 nm (B) and photograph (C) of RR-AuNPs at different times. Figure S9. The UV-Vis absorption spectra (A), the absorbance trends at 530 nm (B) and photograph (C) of RR-AuNPs at different temperatures. Figure S10. The UV-Vis absorption spectra (A), the absorbance trends at 530 nm (B) and photograph (C) of RR-AuNPs at different salt concentrations.
