# Supplementary material for: Green Synthesis and Characterization of Rosa roxburghii Tratt.-Mediated Gold Nanoparticles for Visual Colorimetric Assay of Tiopronin
Source: Nanomaterials (Basel). 2025 Oct 3;15(19):1513. doi: 10.3390/nano15191513 (PMC12525941; doi:10.3390/nano15191513)
Supplement: Supplementary file 1 [file nanomaterials-15-01513-s001.zip › nanomaterials-3827317-supplementary.pdf]

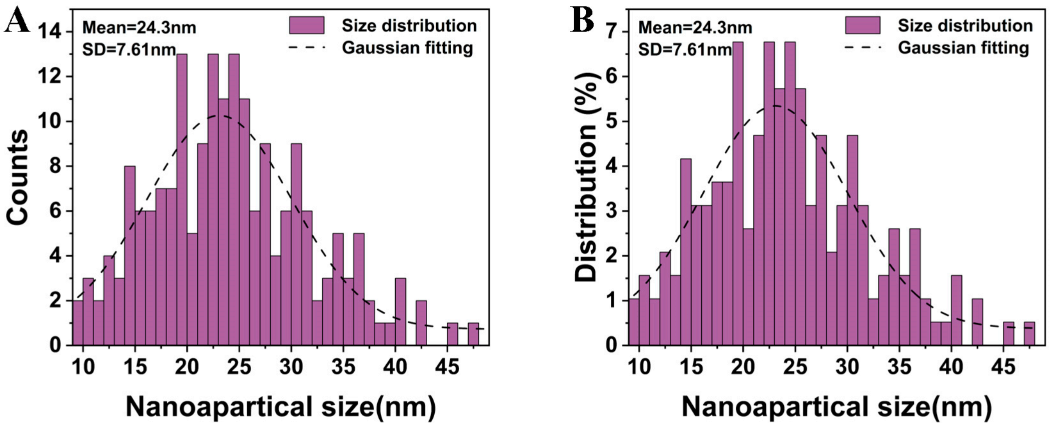


Figure S1. Size distribution histograms of RR-AuNPs from TEM images. (A) Count - based size distribution histogram with Gaussian fitting. (B) Percentage - based size distribution histogram with Gaussian fitting.

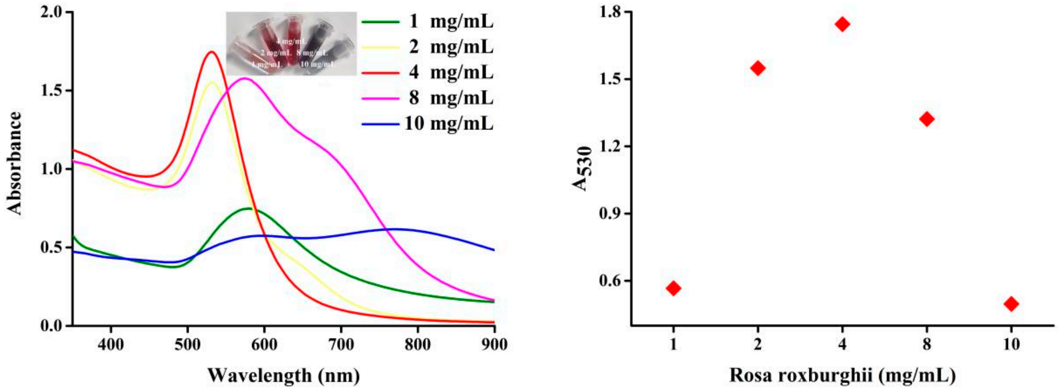


(**A**) (**B**)

Figure S2. The UV-Vis absorption spectra and photograph (A) and the absorbance trends at 530 nm (B) for RR-AuNPs with different concentrations of reducing agent.

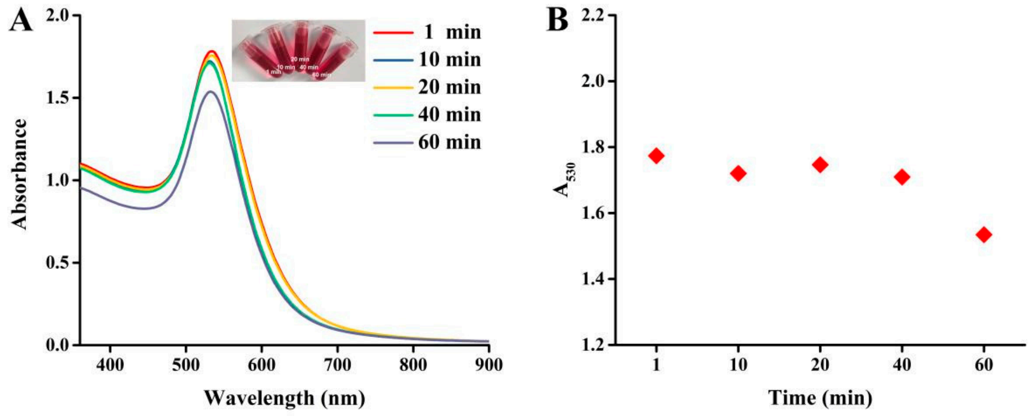


Figure S3. The UV-Vis absorption spectra and photograph (A) and the absorbance trends at 530 nm (B) of RR-AuNPs at different reaction times.

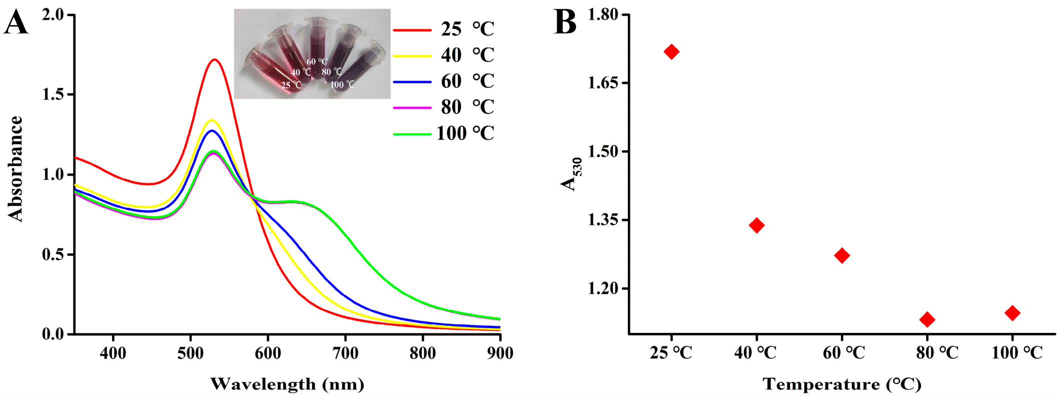


Figure S4. The UV-Vis absorption spectra and photograph (A) and the absorbance trends at 530 nm (B) of RR-AuNPs at different reaction temperatures.

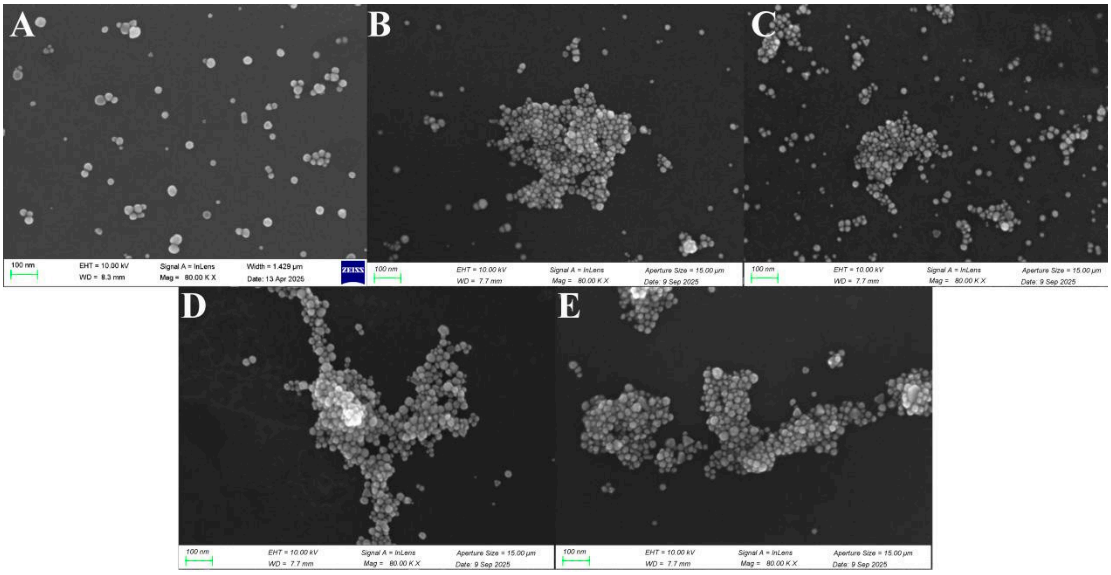


Figure S5. The SEM image of RR-AuNPs at 25℃ (A), at 40℃ (B), at 60℃ (C), at 80℃ (D) and at 100℃(E).

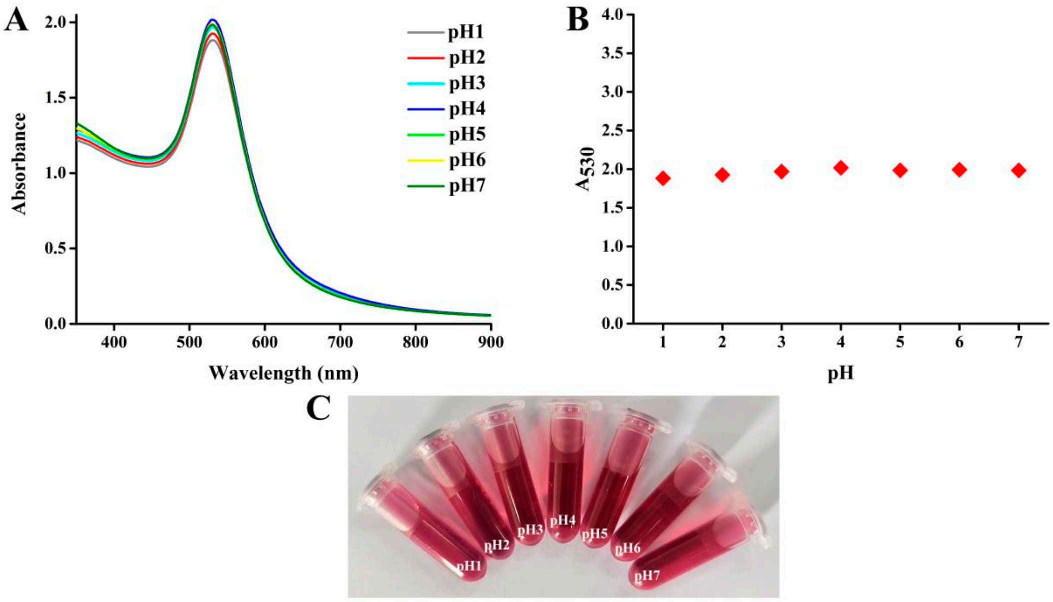


Figure S6. The UV-Vis absorption spectra (A), the absorbance trends at 530 nm (B) and photograph (C) of RR-AuNPs at pH 1-7.

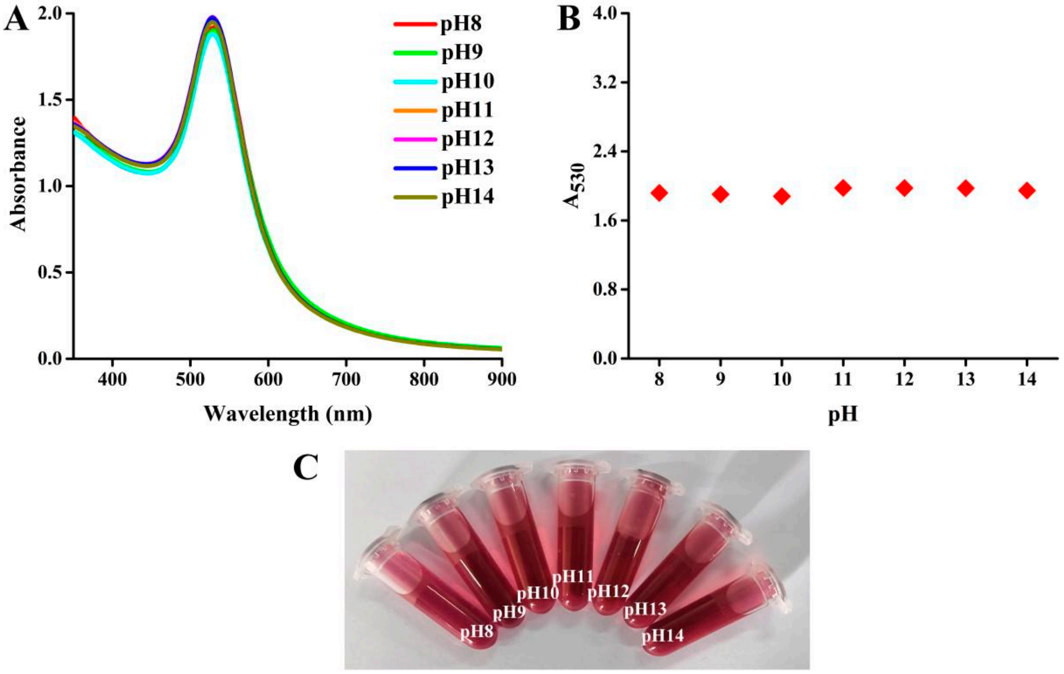


Figure S7. The UV-Vis absorption spectra (A), the absorbance trends at 530 nm (B) and photograph (C) of RR-AuNPs at pH 8-14.

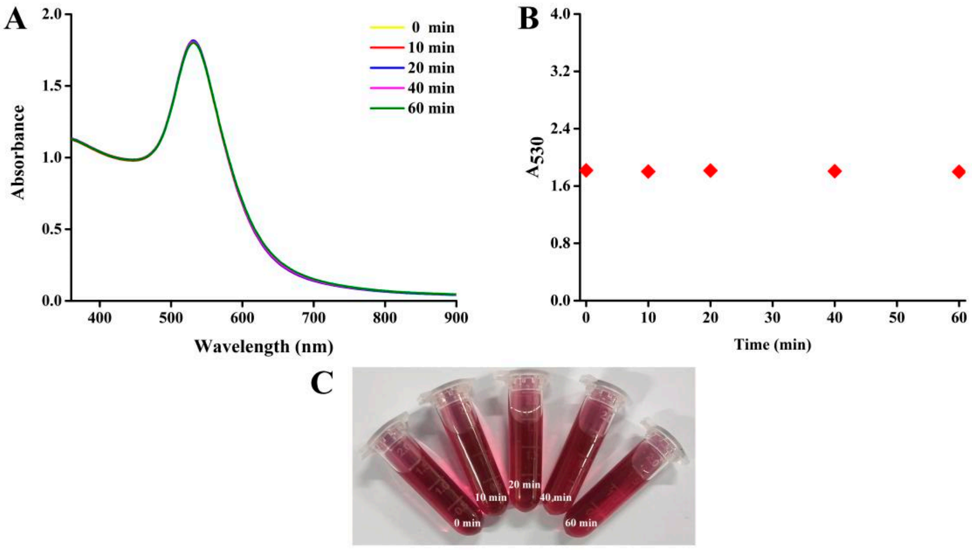


Figure S8. The UV-Vis absorption spectra (A), the absorbance trends at 530 nm (B) and photograph (C) of RR-AuNPs at different times.

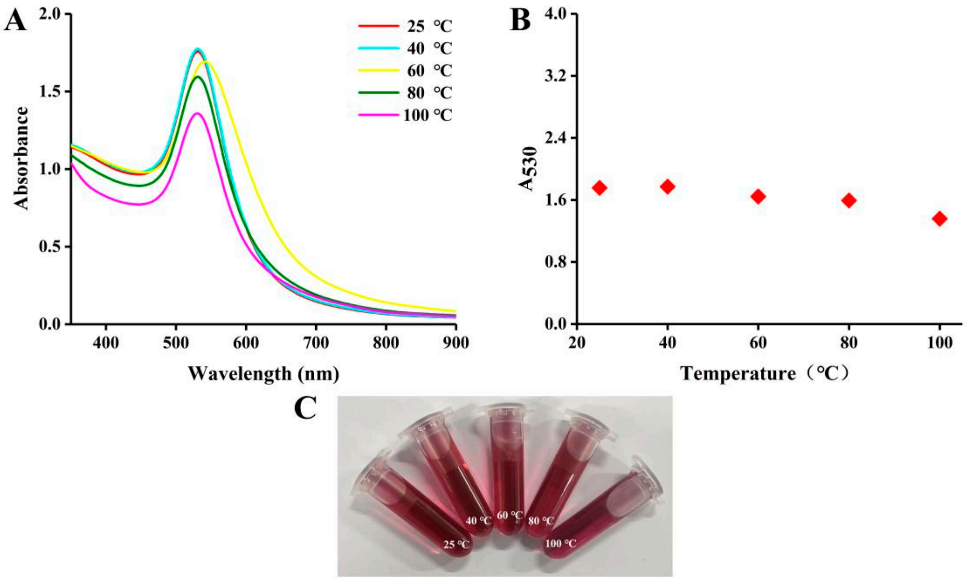


Figure S9. The UV-Vis absorption spectra (A), the absorbance trends at 530 nm (B) and photograph (C) of RR-AuNPs at different temperatures.

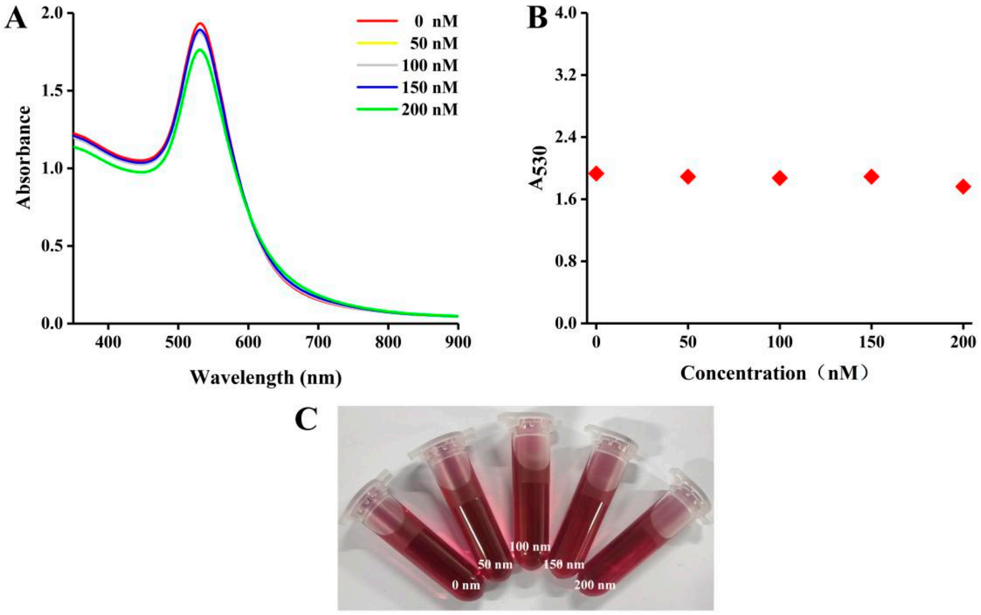


Figure S10. The UV-Vis absorption spectra (A), the absorbance trends at 530 nm (B) and photograph (C) of RR-AuNPs at different salt concentrations.
